# Supplementary material for: Loss of microglial SIRPα promotes synaptic pruning in preclinical models of neurodegeneration
Source: Nat Commun. 2021 Apr 1;12:2030. doi: 10.1038/s41467-021-22301-1 (PMC8016980; doi:10.1038/s41467-021-22301-1)
Supplement: Supplementary file 1 — Supplementary Information [file 41467_2021_22301_MOESM1_ESM.pdf]

Supplementary Figures

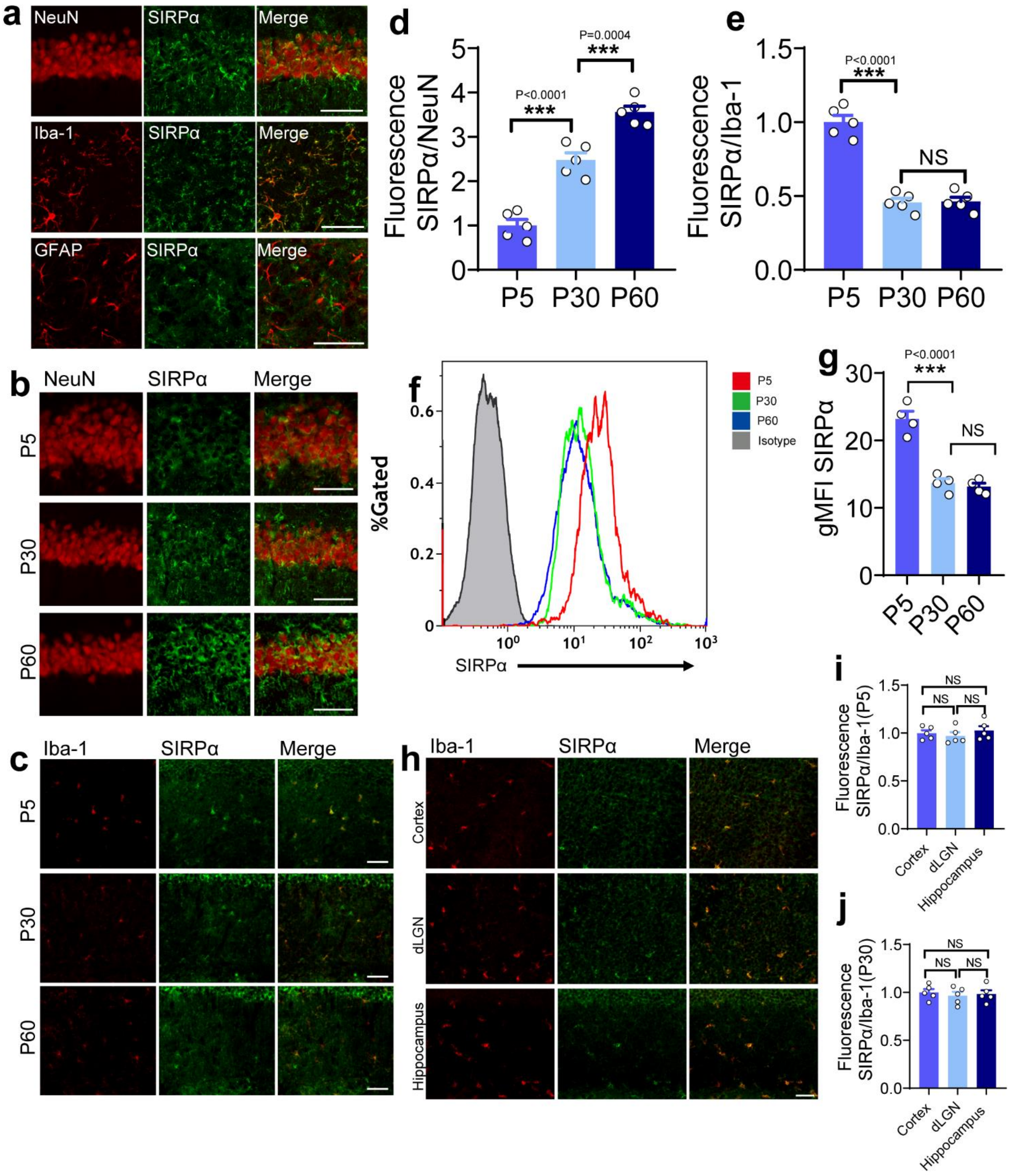

Supplementary Figure 1. Microglial and neuronal SIRPα expression during development stages. **a** SIRPα primarily expresses in microglia (Iba-1$^+$ cell) and neuron (NeuN$^+$ cell) while it is not expressed in astrocyte (GFAP$^+$ cell). Scale bar, 20 μm. This experiment was repeated independently for 3 times. **b, d** Fluorescent images and quantification show that neuronal SIRPα increases during developmental stages (P5, P30, P60). Scale bar, 50 μm. Histogram depicts the relative fluorescent

intensity of SIRPα/NeuN. n=5 mice/group, average of 5-6 fields from each mouse. One-way ANOVA analysis with Dunnett's multiple comparisons test. **c, e** Fluorescent images and quantification show that SIRPα expression in microglia decreases from P5 to P30, remaining unchanged from P30 to P60. Scale bar, 50 μm. Histogram depicts the relative fluorescent intensity of SIRPα/Iba-1. n=5 mice/group, average of 5-6 fields from each mouse. One-way ANOVA analysis with Dunnett's multiple comparisons test. **f, g** Flow cytometry analysis shows microglial SIRPα in mice brain decreases from P5 to P30, remaining unchanged from P30 to P60. Histogram shows the gMFI quantification of microglial SIRPα. n= 4 mice/group, average of 3 tests from each mouse. One-way ANOVA analysis with Dunnett's multiple comparisons test. **h** Representative images of P5 WT mice exhibit no difference of microglial SIRPα in cortex, hippocampus and dLGN. Scale bar, 50 μm. **i, j** Histograms show that microglial SIRPα level in cortex, hippocampus and dLGN are comparable in P5 and P30 mice respectively. One-way ANOVA analysis with Tukey's multiple comparisons test. n=5 mice/group. Data are mean ± s.e.m. *** $P < 0.001$, NS, not significant. Detailed statistical information was listed in Supplementary Statistical Data. Source data are provided as a Source Data file.

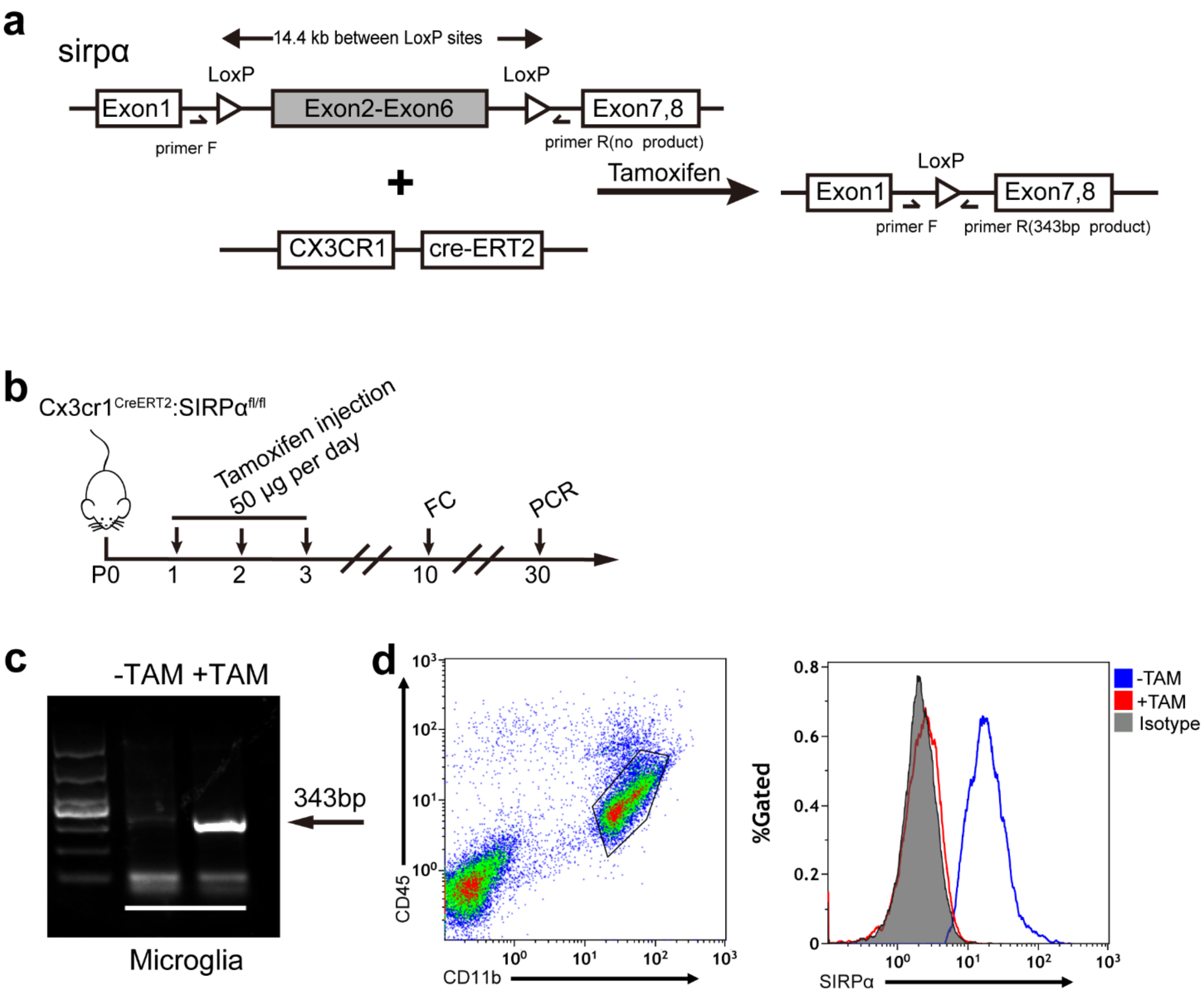

Supplementary Figure 2. Establishment of microglia specific SIRPα knockout mouse line. a, Schematic of generating SIRPα conditional KO mice. SIRPα conditional knockout (SIRPα-cKO) mice were generated by crossing SIRPα$^{fl/fl}$ mice, in which exon 2-6 of Sirpα gene was flanked by LoxP sites, with Cx3cr1^{CreERT2} mice to obtain Cx3cr1^{CreERT2}:SIRPα $^{fl/fl}$ mice. The length between the two LoxP sites was 14.4kb. **b** Experimental design for testing the efficiency of specific gene deletion in SIRPα-cKO mice. Cx3cr1^{CreERT2}:SIRPα$^{fl/fl}$ mice were injected with tamoxifen at P1-P3 to delete exon 2-6 of sirpa gene in Cx3cr1^{+} cells. **c** PCR was used to amplify DNA extracted from isolated microglia. There is a 343bp fragment after we induce deletion of exon 2-6 with Tamoxifen (TAM). In SIRPα$^{+/+}$ cells (Cx3cr1^{CreERT2}:SIRPα $^{fl/fl}$ without TAM), genetic distance between two primers is longer than 14kb, which produces no products under normal PCR condition (elongation time 45 s). This experiment was repeated independently for 3 times. **d** Left panel, gating strategy used to identify microglia from single-cell suspension prepared from brains of control(-TAM) and SIRPα-cKO(+TAM) mice. CD45^{int} CD11b^{high} cells (population in pentagon) were considered as microglia. This gating strategy also corresponds to FACS data in the manuscript figures (fig.6 c and i; fig.8 f). Right panel, microglia of SIRPα-cKO (+TAM) mice showed scarce SIRPα expression (0.41%) while control (-TAM) mice showed a relatively higher expression (92.76%) of surface SIRPα. This experiment was repeated independently for 3 times.

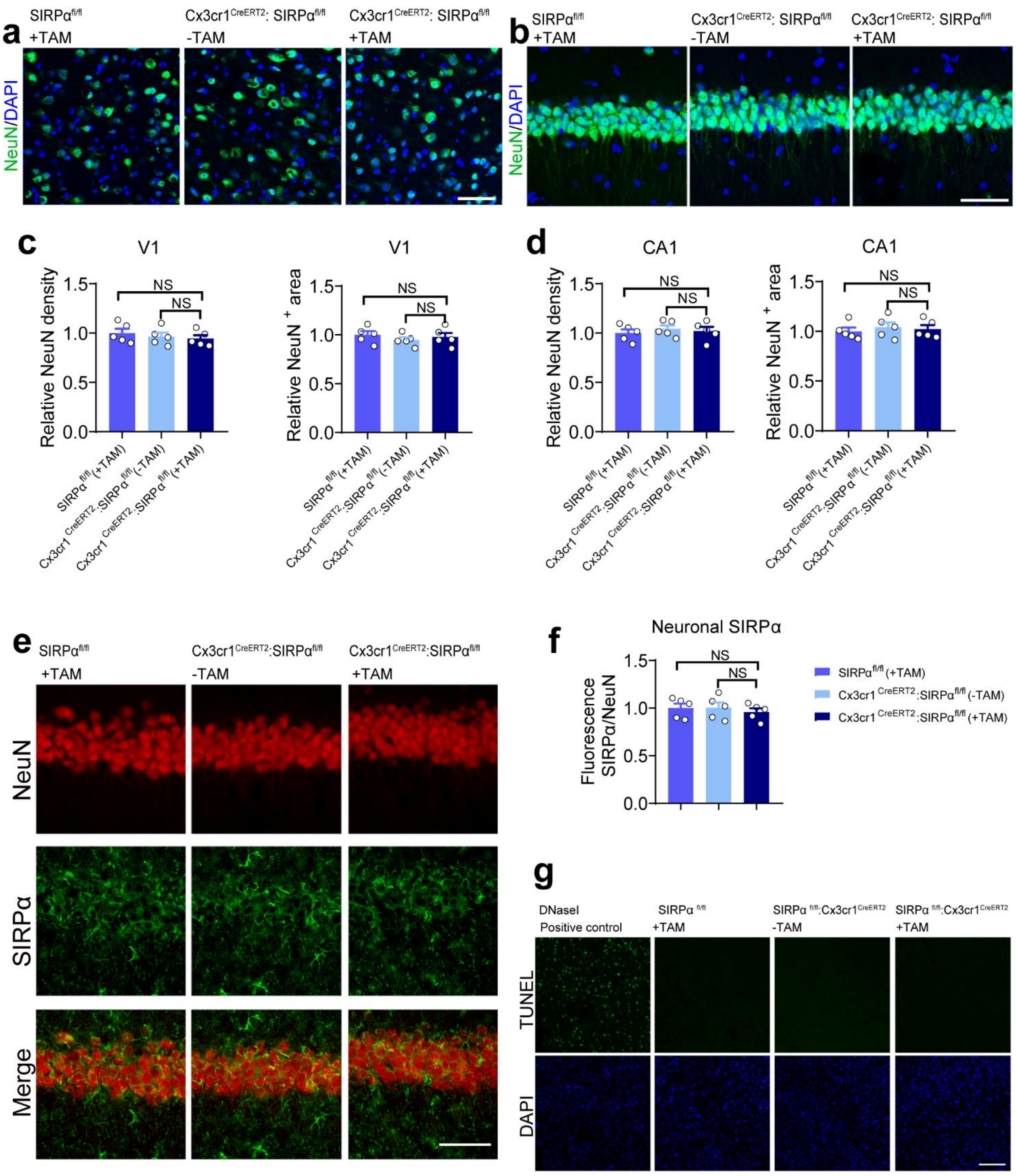

Supplementary Figure 3. Neuronal density, morphology and SIRPα expression in SIRPα-cKO mice. a-d NeuN/DAPI staining demonstrates that neuronal density ($NeuN^+$ cell number/area of interest) and cell volume ($NeuN^+$ area per cell) are not altered in cortex (a, c) and hippocampus (b, d) of SIRPα-cKO mice. Scale bar, 50 μm. n=5 mice/group, average of 5-6 fields from each mouse. One-way ANOVA analysis with Dunnett's multiple comparisons test. **e-f** Immunostaining and quantification show that neuronal SIRPα expression (fluorescent intensity of SIRPα/NeuN) is not changed after

microglial SIRPα deletion. Scale bar, 50 µm. n=5 mice/group, average of 5-6 fields from each mouse. One-way ANOVA analysis with Dunnett's multiple comparisons test. **g** TUNEL staining reveals little apoptotic signal in these mice. DNase I treated brain sections are used as positive control. Scale bar, 100 µm. This experiment was repeated independently for 3 times. Data are mean ± s.e.m. NS, not significant. Detailed statistical information was listed in Supplementary Statistical Data. Source data are provided as a Source Data file.

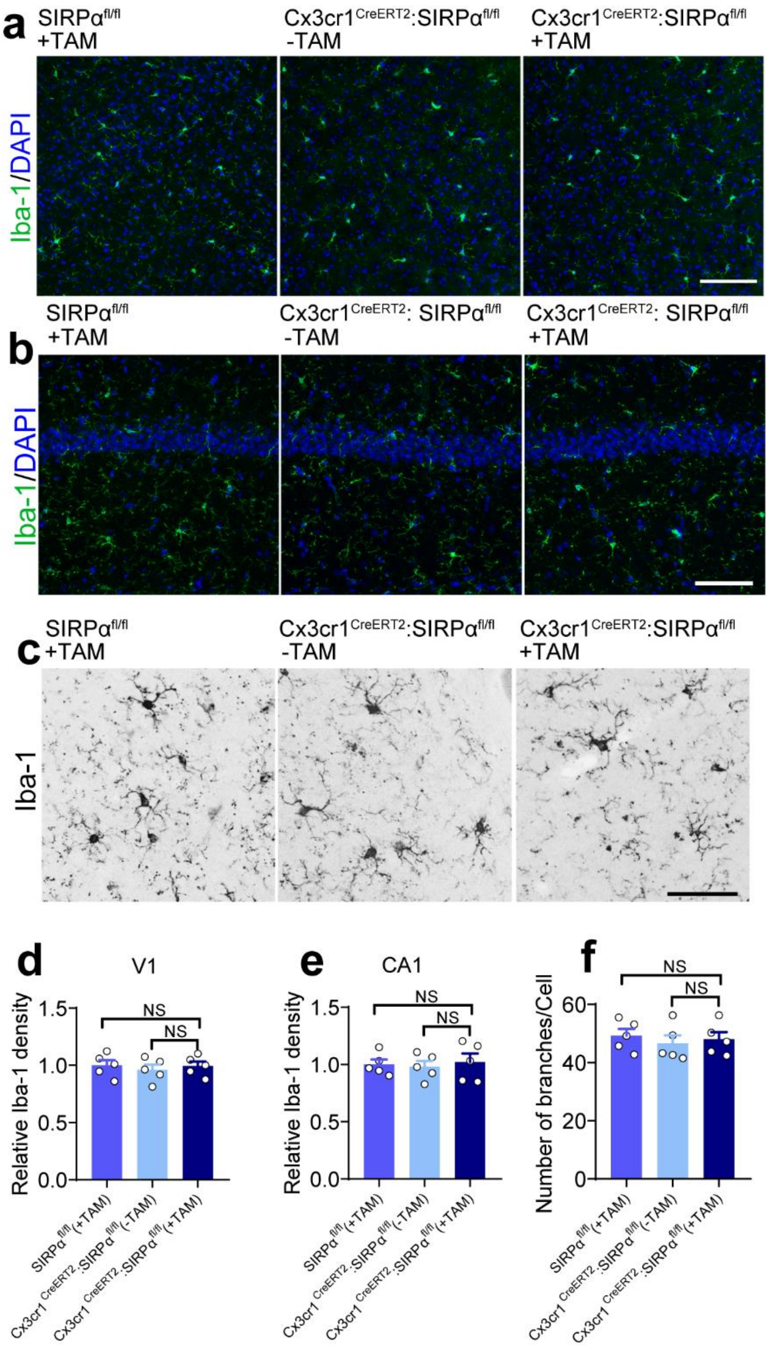

Supplementary Figure 4. Microglial density and morphology in SIRPα-cKO mice. a-b Immunofluorescent images of Iba-1$^+$ microglia in the cortex (a) and hippocampus (b) of SIRPα-cKO and control mice. Scale bar, 100 μm. **c** Iba-1 fluorescent images are performed z-stack projection (at 1 μm intervals) and converted into 8-bit. Scale bar, 50 μm. **d-e** Histograms depict microglial density (Iba-1$^+$ cell number/area of interest) are not altered in SIRPα-cKO mice. n=5 mice/group, average of 5-6 fields from each mouse. One-way ANOVA analysis with Dunnett's multiple comparisons test. **f** Histogram shows microglial morphology (branches number per cell) is not significantly changed in SIRPα-cKO mice. n=5 mice/group, average of 5-6 fields from each mouse. One-way ANOVA analysis with Dunnett's multiple comparisons test. Data are mean ± s.e.m. NS, not significant. Detailed statistical information was listed in Supplementary Statistical Data. Source data are provided as a Source Data file.

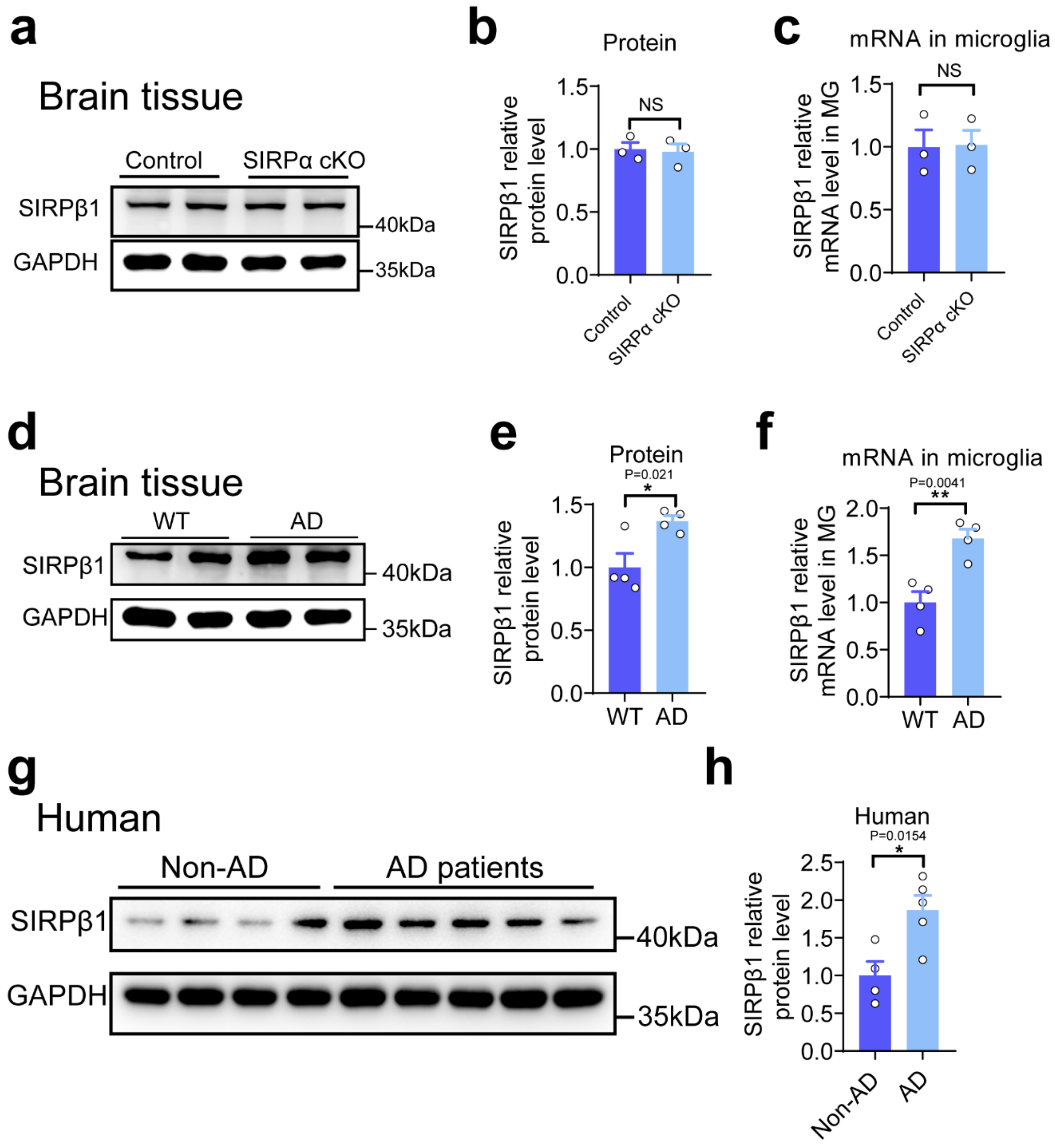

Supplementary Figure 5. SIRPβ1 expression in SIRPα-cKO mice, AD mice and AD patients. a-c Western blot (a) and quantification (b) show that SIRPβ1 protein level is not altered in SIRPα-cKO mice brain; (c) SIRPβ1 mRNA level in microglia remains unchanged after SIRPα ablation, n=3 mice/group, two-tailed unpaired t-test. (Primers used for quantification are listed in Supplementary Table 1.) **d-f** Western blot (d) and quantification (e) show that SIRPβ1 protein level increase in 8-months old AD mice brain; (f) microglial SIRPβ1 mRNA level is upregulated in 8-months old AD mice brain. n=4 mice/group, two-tailed unpaired t-test. **g-h** Western blot (g) and quantification (h) show that SIRPβ1 protein level is significantly increased in cortex of AD patients. Non-AD=4, AD patients=5. Two-tailed unpaired t test. Detailed sample information is listed in the table 1 in methods section. Data are mean ± s.e.m. * $P < 0.05$, ** $P < 0.01$, NS, not significant. Detailed statistical information was listed in Supplementary Statistical Data. Source data are provided as a Source Data file.

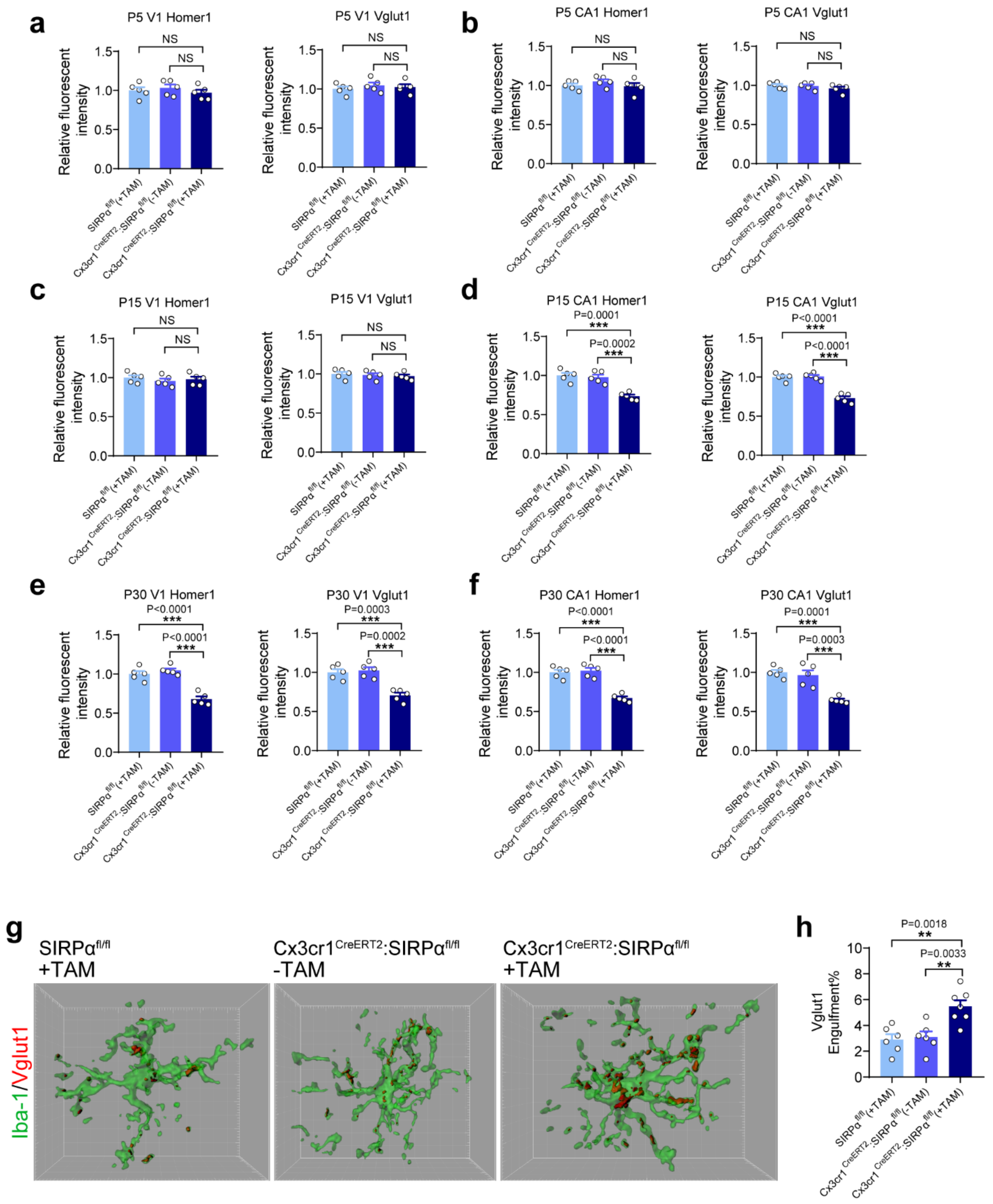

Supplementary Figure 6. Quantification of synaptic density and microglial engulfment in SIRPα-cKO mice (Cx3cr1^{CreERT2}:SIRPα$^{fl/fl}$ (+TAM)). a-f Histograms depict the statistics of Vglut1 or Homer1 density in V1 (a, c e) or hippocampal CA1 (b, d, f) in SIRPα$^{fl/fl}$ (+TAM), Cx3cr1^{CreERT2}:SIRPα$^{fl/fl}$ (-TAM) and Cx3cr1^{CreERT2}:SIRPα$^{fl/fl}$ (+TAM) mice at different timepoints. n=5 mice/group; average of 10-12 fields from each mouse. One-way ANOVA analysis with Dunnett's multiple comparisons test. **g, h** 3D reconstruction and surface rendering in V1 demonstrate that Vglut1 puncta volume in Iba-1 positive microglia from SIRPα-cKO mice was larger than control mice. Grid line increments= 5 μm. n=6, 6, 7 mice/group, average of 8-9 microglia from each mouse, one-way

ANOVA analysis with Dunnett's multiple comparisons test. Data are mean ± s.e.m. ** $P < 0.01$, *** $P < 0.001$, NS, not significant. Detailed statistical information was listed in Supplementary Statistical Data. Source data are provided as a Source Data file.

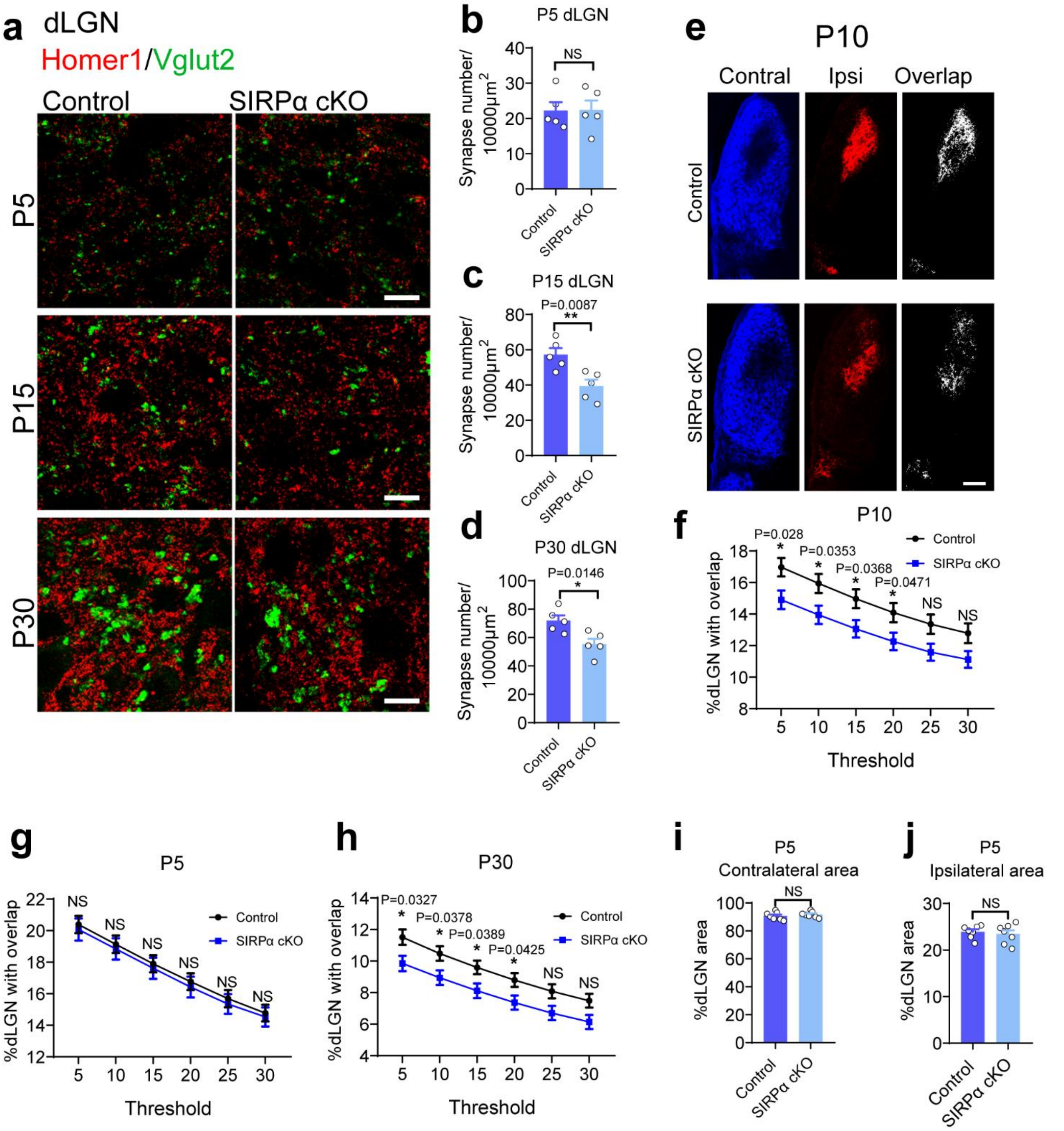

Supplementary Figure 7. Synaptic density and eye-specific segregation in dLGN of SIRPα-cKO mice. a-d Representative images and quantification of synaptic number in dorsal lateral geniculate nucleus (dLGN) of SIRPα-cKO and control mice at different timepoints (Cx3cr1^{CreERT2}:SIRPα$^{fl/fl}$ (-TAM) are used as normal control). Synaptic number was quantified as colocalized presynaptic marker Vglut2 (green) and postsynaptic marker Homer1 (red). Scale bar, 10 μm. n=5 mice/group; average of 6-8 fields from each mouse. Two-tailed unpaired t-test. **e-h** Retinal ganglion cell (RGC) inputs to dLGN was labeled by intraocular injection of cholera toxin subunit B-594 (CTB-594) and CTB-647 into two eyes respectively. Representative images show dLGN contralateral (blue) and ipsilateral (red) territories of SIRPα-cKO and control mice at P10(e). Scale bar, 100 μm. Quantification of eye

segregation shows SIRPα-cKO mice exhibit less overlap between the two territories at P10(f) and P30(h), while it remains unchanged at P5(g). n=7 mice/group, two-tailed unpaired t-test. **i-j** The average area of the contralateral or ipsilateral patch are not significantly altered in P5 SIRPα-cKO mice compared to control, indicating similar neural innervation. n=7 mice/group; two-tailed unpaired t-test. Data are mean ± s.e.m. * $P < 0.05$, ** $P < 0.01$, NS, not significant. Detailed statistical information was listed in Supplementary Statistical Data. Source data are provided as a Source Data file.

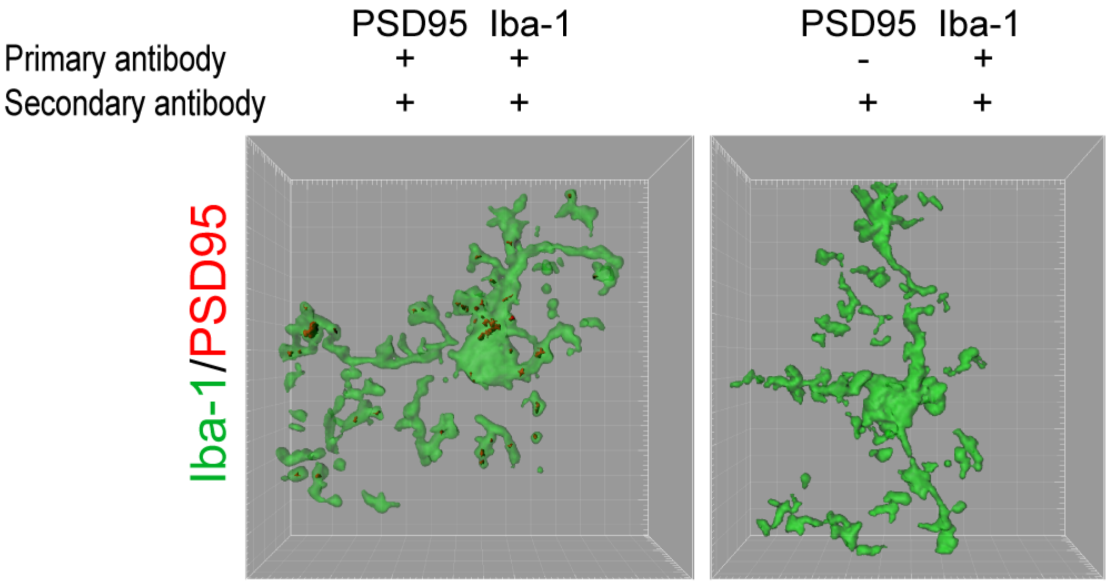

Supplementary Figure 8. Brain section stained with secondary antibody only for PSD95 (right panel) demonstrated the specificity of synaptic marker labeling. Grid line increments= 5 μm.

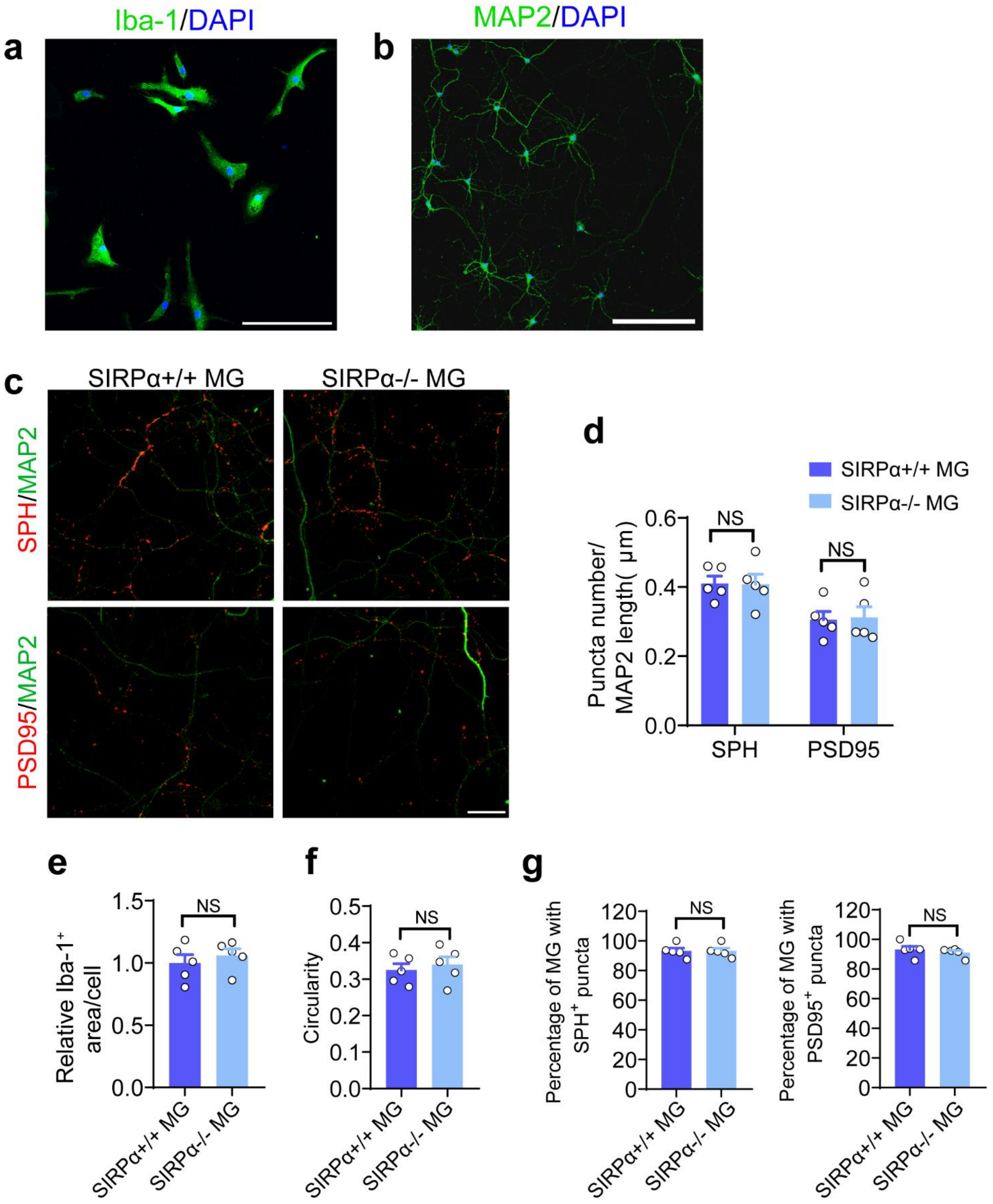

Supplementary Figure 9. Morphological analysis of primary cell culture. a Iba-1/DAPI staining reveals high purity (>90%) of primary microglia before co-culture. Scale bar, 100 μm. This experiment was repeated independently by 3 times. **b** MAP2/DAPI staining reveals high purity (>90%) of primary neuron before co-culture. Scale bar, 200 μm. This experiment was repeated independently by 3 times. **c-d** Representative images and quantification show the overall synaptic density (PSD95 or SPH) in areas absent of microglia are not altered. Scale bar, 20 μm, n=5

independent experiment, average 5-6 fields from each assay, two-tailed unpaired t-test. MG, microglia; SPH, Synaptophysin. **e-f** Area and circularity of SIRPα$^{+/+}$ and SIRPα$^{-/-}$ microglia in co-culture are comparable. n=5 independent experiment, average 5-6 fields from each assay. Two-tailed unpaired t-test. **g** Percentage of microglia that contained PSD95/SPH positive structures are equivalent in both groups. n=5 independent experiment, average 5-6 fields from each assay, two-tailed unpaired t-test. Data are mean ± s.e.m. NS, not significant. Detailed statistical information was listed in Supplementary Statistical Data. Source data are provided as a Source Data file.

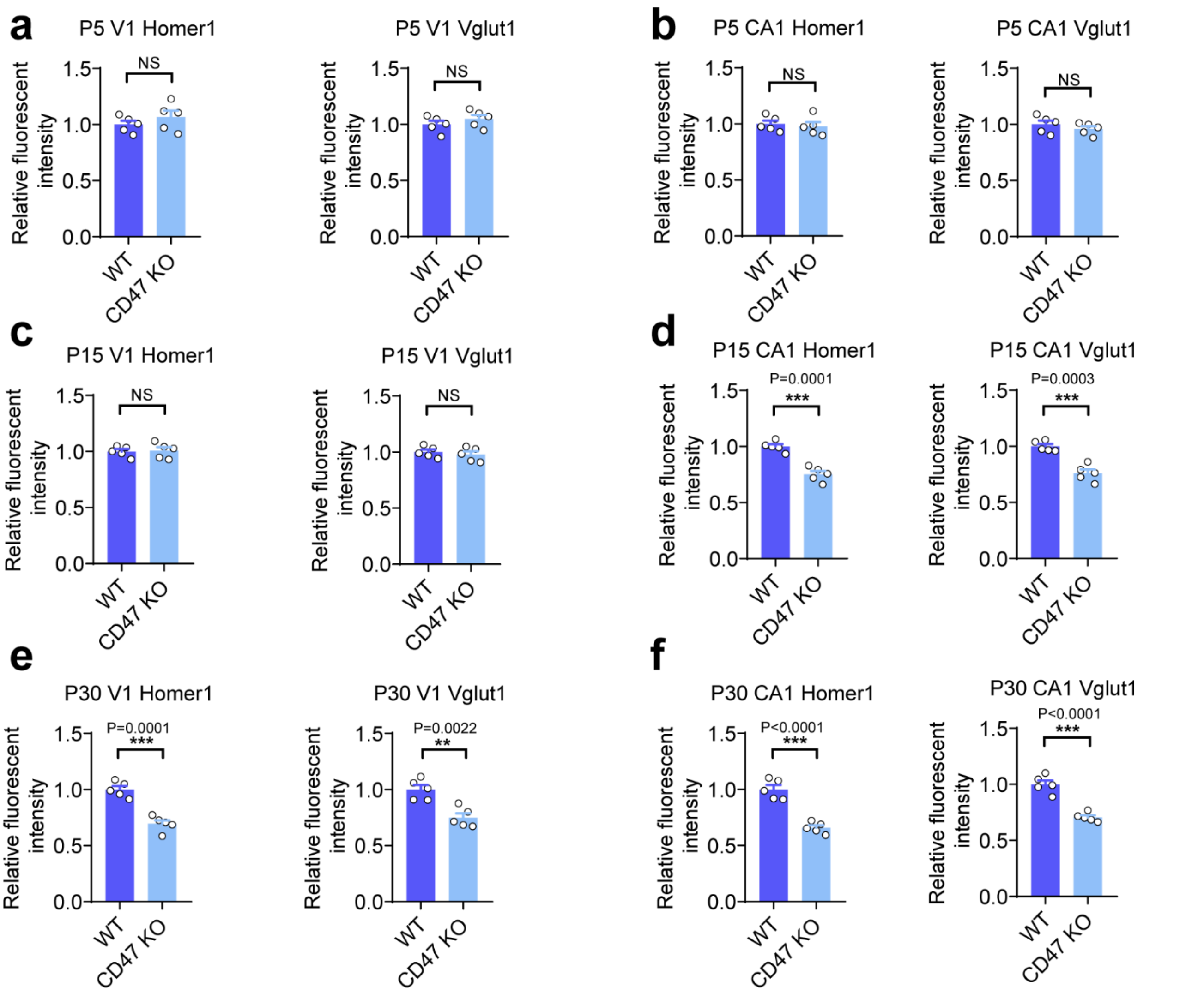

Supplementary Figure 10. Synaptic density in CD47-KO mice is lower than WT mice. a-f Histograms depict the statistics of Vglut1 and Homer1 density in V1 cortex (a, c, e) or hippocampal CA1 (b, d, f) in WT and CD47-KO mice at different timepoints. n=5 mice, 10-12 fields from each mouse. Two-tailed unpaired t-test. Data are mean ± s.e.m. ** $P < 0.01$, *** $P < 0.001$, NS, not significant. Detailed statistical information was listed in Supplementary Statistical Data. Source data are provided as a Source Data file.

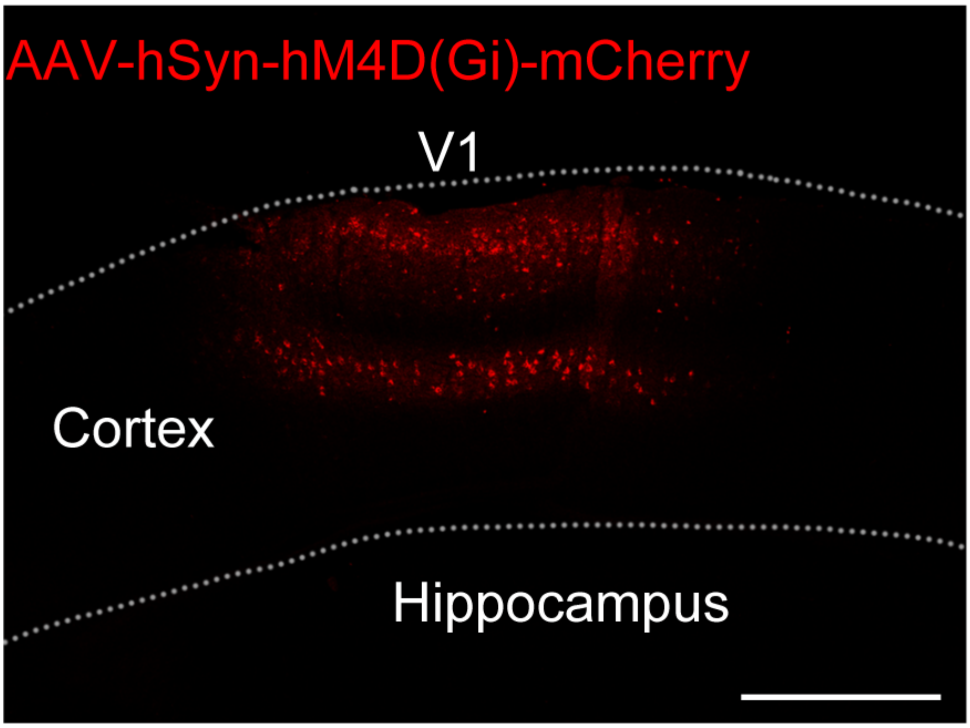

Supplementary Figure 11. Fluorescent signal (mCherry, red) in cortex 4 weeks after the injection of AAV-hSyn-hM4D(Gi)-mCherry, scale bar, 500μm. This experiment was repeated independently for 5 times.

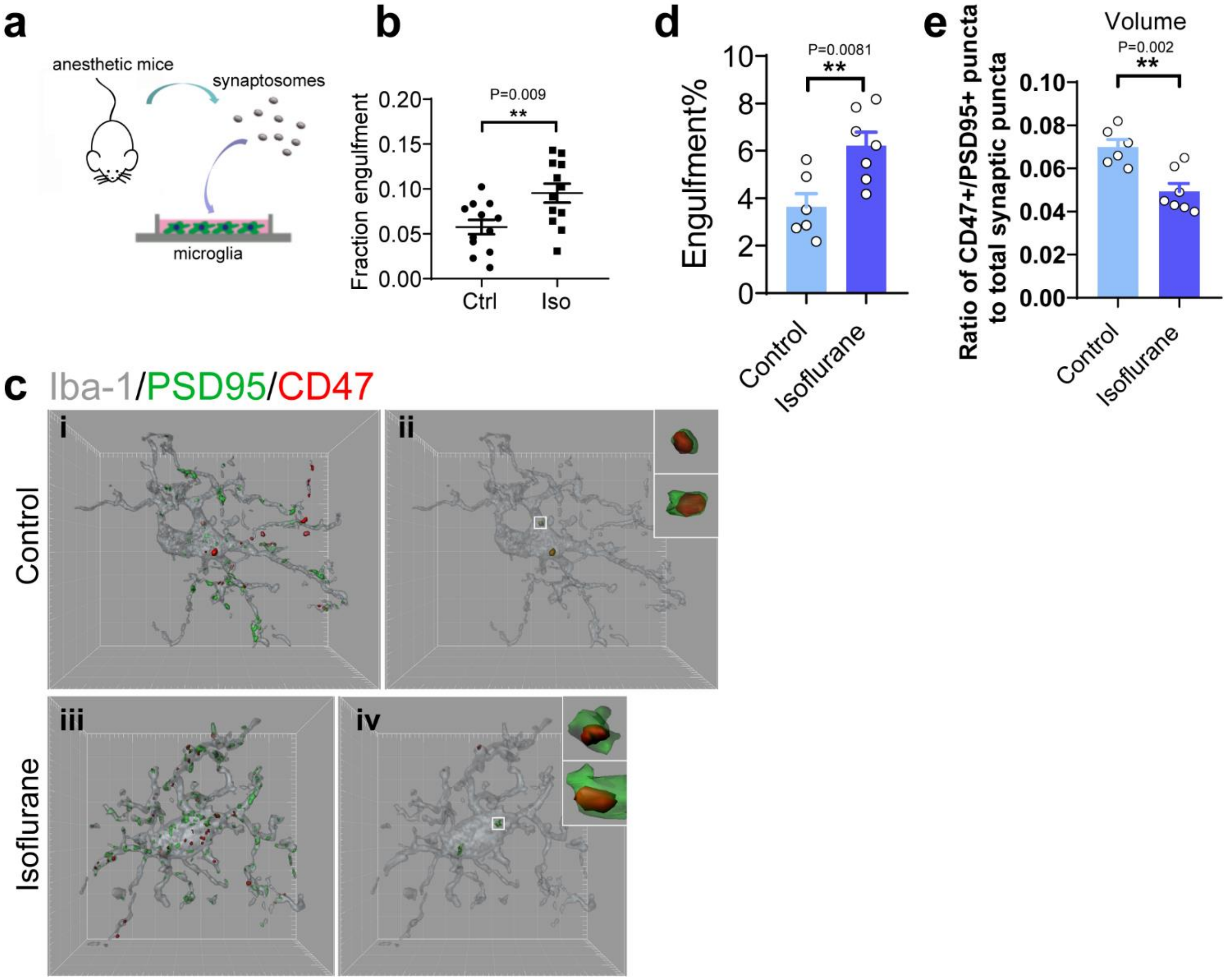

Supplementary Figure 12. Microglia engulfed more synaptic structures from anesthetic mice. a-b Diagram (a) shows that synaptosomes isolated from anesthetic mice were added into microglia. Phagocytic analysis (b) shows that microglia preferred to engulf synaptosomes from isoflurane treated mice rather than those from control mice. n=12 wells; average of 5-6 fields from each well, two-tailed unpaired t test. **c** Left panels show CD47^{+} or PSD95^{+} signal inside microglia from anesthetic and control mice (i and iii); right panels (ii and iv) show CD47 and PSD95 colocalized puncta inside cells. Insets are enlarged images with orthogonal plane of typical CD47^{+}/PSD95^{+} puncta. Grid line increments= 5 µm. **d** Statistic analysis reveals larger volume ratio of PSD95 puncta inside Iba-1 positive microglia from anesthetic mice, n=6, 7 mice/group, average of 8-9 microglia from each mouse, two-tailed unpaired t test. **e** Histogram shows volume ratio of CD47^{+}/PSD95^{+} puncta to total synaptic structures in control and anesthetic mice, n=6, 7 mice/group, average of 8-9 cells from each mouse, two-tailed unpaired t test. Data are mean ± s.e.m. ** $P < 0.01$. Detailed statistical information was listed in Supplementary Statistical Data. Source data are provided as a Source Data file.

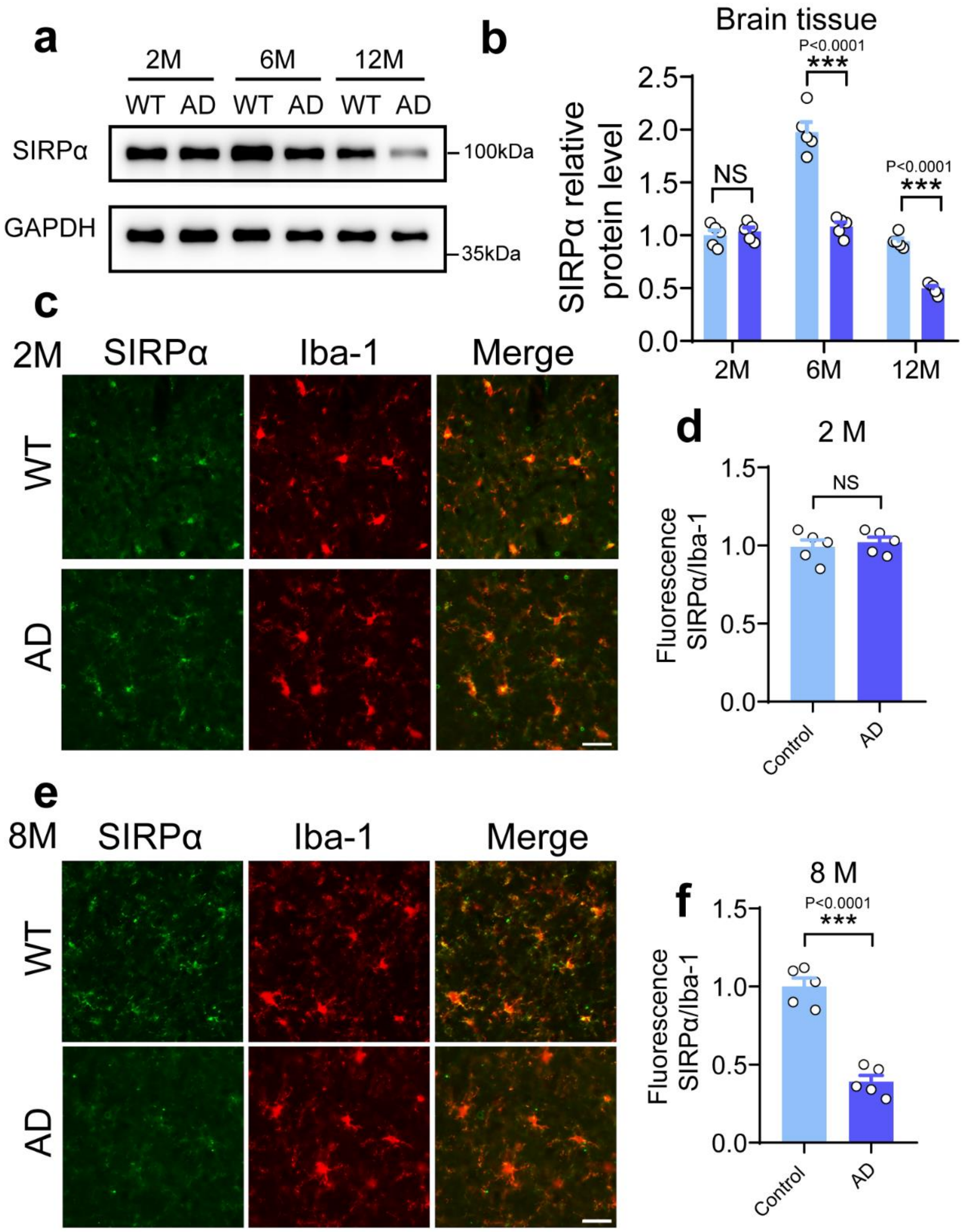

Supplementary Figure 13. Microglial SIRPα expression decreases in AD mice. a-b Western blot and statistical analysis of SIRPα expression in 2-months, 6-months and 12-months old WT and AD mice brain. n=5 mice/group, two-way ANOVA via Sidak's multiple comparisons test. **c-d** Immunostaining and quantification of SIRPα with microglial marker (Iba-1) in cortex of WT and AD mice (2-months old). Scale bar, 25μm, n=5 mice/group; average of 8 fields from each mouse, two-tailed unpaired t test. **e-f** Immunostaining and quantification of SIRPα with microglial marker (Iba-1) in cortex of WT and AD mice (8-months old). Scale bar, 25μm, n=5 mice/group; average of 8 fields from each mouse, two-tailed unpaired t test. Data are mean ± s.e.m. *** $P < 0.001$, NS, not significant. Detailed statistical information was listed in Supplementary Statistical Data. Source data are provided as a Source Data file.

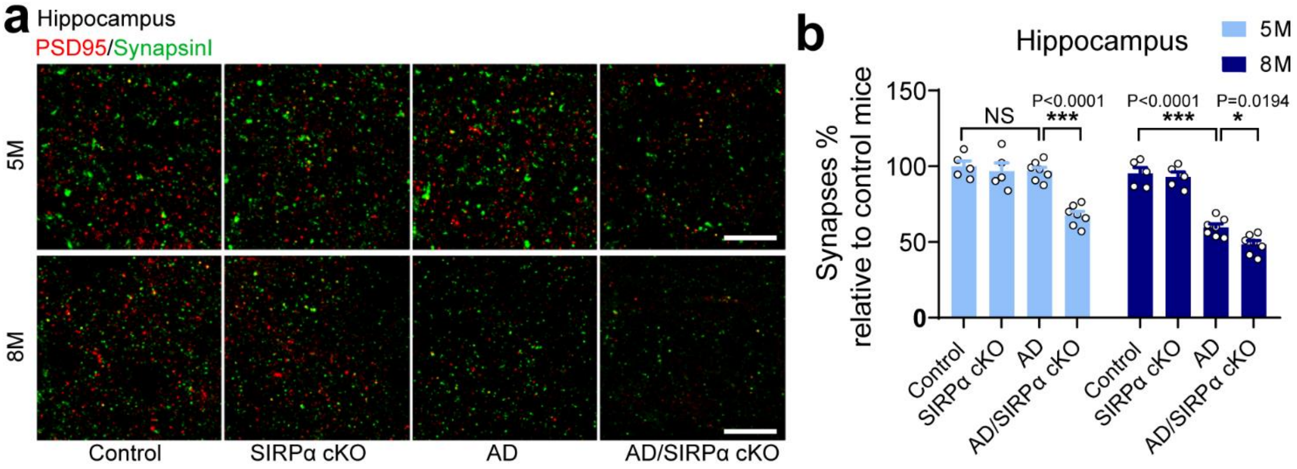

Supplementary Figure 14. Synaptic density in hippocampus of 5-months old and 8-months old mice of 4 different genotypes. a, b Representative images and quantification of synapses staining for presynaptic marker Synapsin I (green) and postsynaptic marker PSD95 (red) in hippocampus of 5-months old and 8-months old mice. Synaptic number was determined as colocalized pre- and post-synaptic puncta. Scale bars, 10 μm. n=5, 5, 7, 7 mice/group, average of 10-12 fields from each mouse, one-way ANOVA, via Dunnett's multiple comparisons test. Data are mean ± s.e.m. * $P < 0.05$, *** $P < 0.001$, NS, not significant. Detailed statistical information was listed in Supplementary Statistical Data. Source data are provided as a Source Data file.

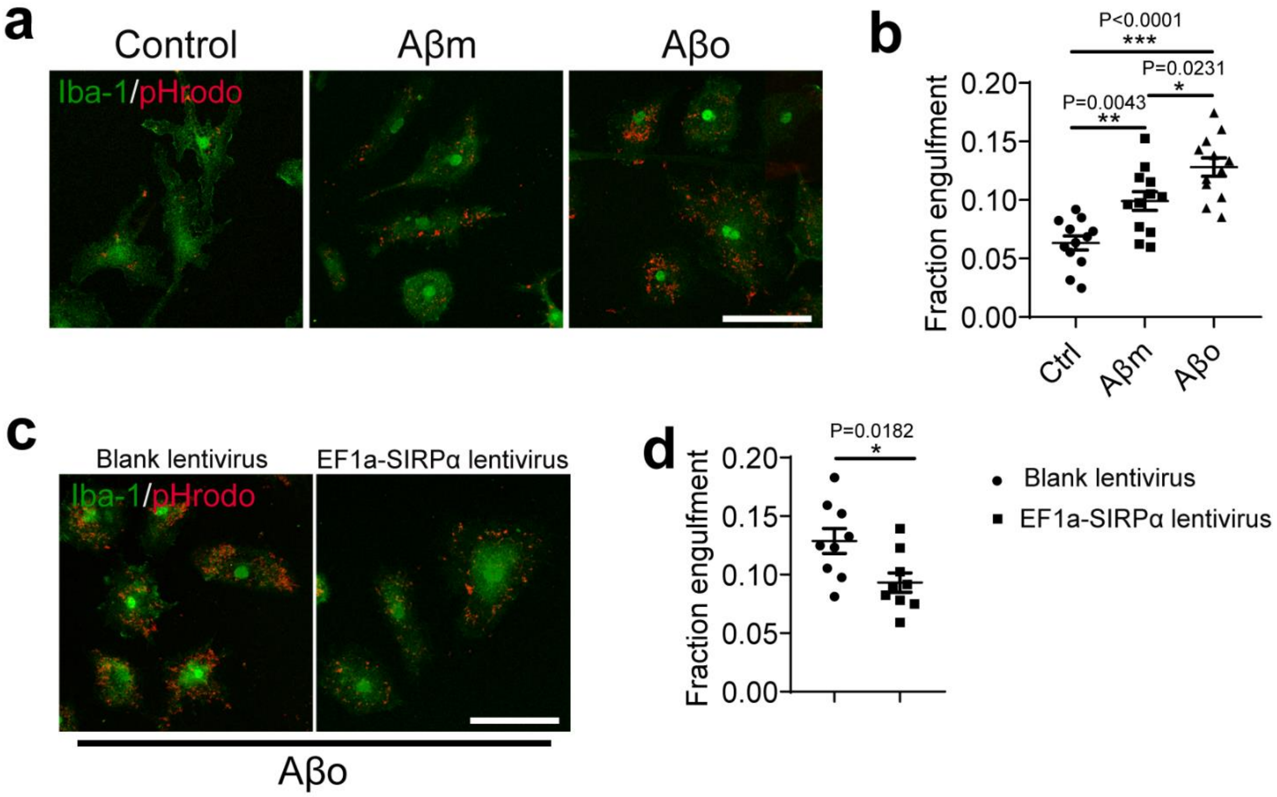

Supplementary Figure 15. Aβ stimulation increases microglial engulfment of synaptosomes that is rescued by SIRPα overexpression a, b Representative images (a) and quantification (b) show that Aβ treatment (0.2 μM for 3h) increases microglial engulfment of pHrodo-conjugated synaptosomes. Scale bar, 75μm. Aβm, $A\beta_{42}$ monomer; Aβo, $A\beta_{42}$ oligomer. n=12 wells, average of 5-6 fields from each well; one-way ANOVA via Tukey's multiple comparisons test. **c, d** Representative images (c) and quantification (d) show that SIRPα overexpression alleviates microglial phagocytosis of pHrodo-conjugated synaptosomes induced by Aβo treatment. Scale bar, 75μm. n=9 wells, average of 5-6 fields from each well; two-tailed unpaired t-test. Data are mean ± s.e.m. * $P < 0.05$, ** $P < 0.01$, *** $P < 0.001$. Detailed statistical information was listed in Supplementary Statistical Data. Source data are provided as a Source Data file.

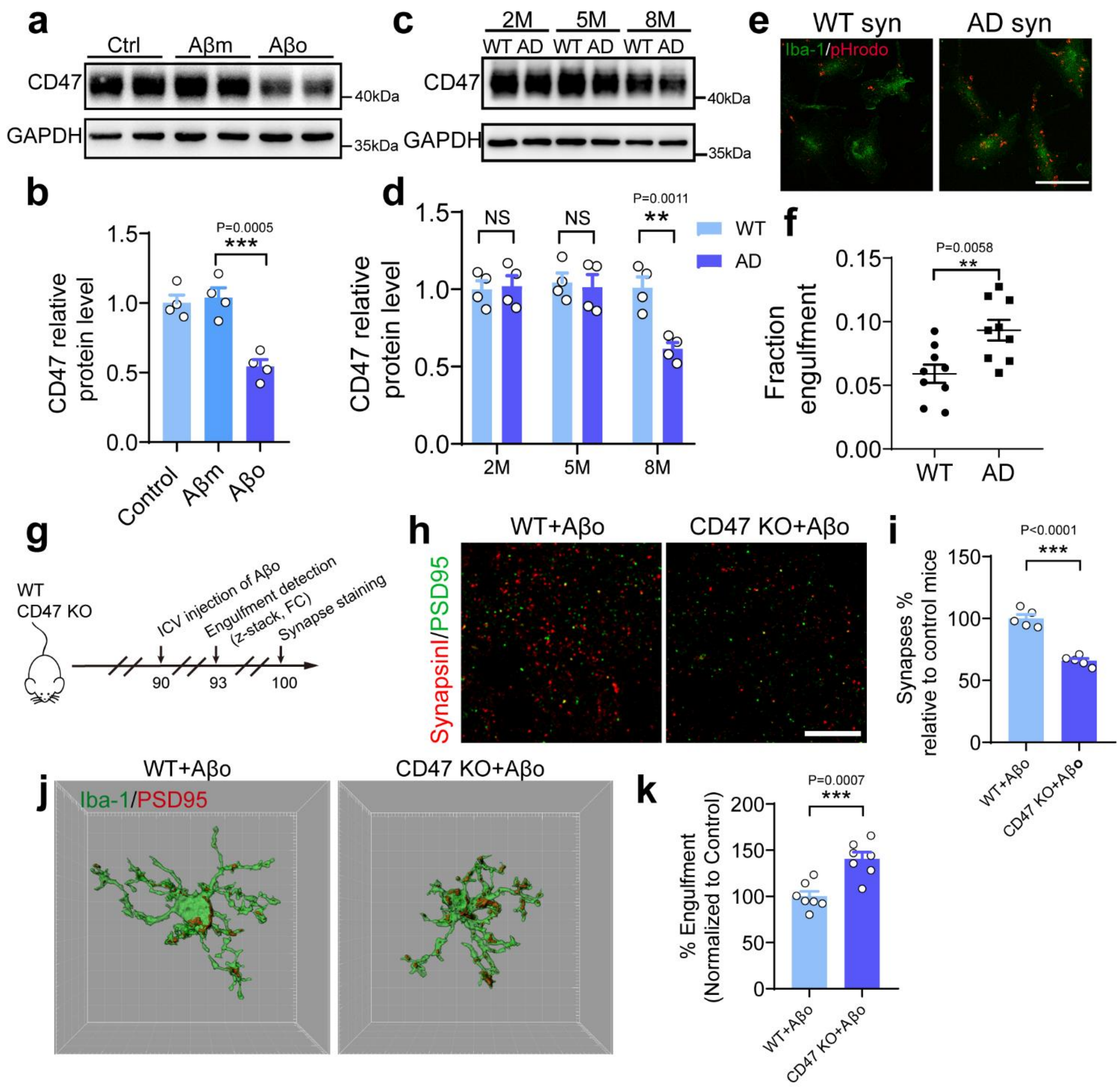

Supplementary Figure 16. CD47 deficiency facilitates synaptic loss in mice after Aβo administration. a-b Western blot and statistical analysis of CD47 protein level in primary cultured neuron after $A\beta_{42}$ monomer (Aβm) or $A\beta_{42}$ oligomer (Aβo) treatment. Cells were treated with 0.2 μM Aβ for 24 h before protein analysis. n=4 experiments, one-way ANOVA via Dunnett's multiple comparisons test. **c-d** Western blot and statistical analysis of CD47 expression in synaptosome from 2-months, 5-months and 8-months old WT and AD mice brain. n=4 mice/group, two-way ANOVA via Sidak's multiple comparisons test. **e-f** Phagocytic assay shows that synaptosomes (Syn) derived from 8-months old AD mice are inclined to be engulfed by microglia. Scale bar, 75μm. n=9 wells, average of 5-6 fields from each well; two-tailed unpaired t test. **g** Schematics of the experimental procedures. ICV, intracerebroventricular; FC, Flow cytometry. **h-i** Representative images (h) depict synaptic staining for presynaptic marker Synapsin I (red) and postsynaptic marker PSD95 (green) in cortex. The histogram (i) displays the quantification of synaptic density in CD47KO and control mice after Aβo ICV injection (2 μg/mouse). Scale bar, 10 μm. n=5 mice/group (male, 3-months age), average of

6-8 fields from each mouse, two-tailed unpaired t-test. **j-k** 3D reconstruction and surface rendering demonstrate larger volumes of $PSD95^+$ puncta inside Iba-1^+ microglia in cortex from CD47 deficient mice versus control mice after Aβo stimulation. n=7 mice/group, average of 8-9 microglia from each mouse, two-tailed unpaired t test. Grid line increments, 5 μm. Data are mean ± s.e.m. ** $P < 0.01$, *** $P < 0.001$, NS, not significant. Detailed statistical information was listed in Supplementary Statistical Data. Source data are provided as a Source Data file.

Supplementary Table 1 The sequences of primers used in the study.

**Mice and PCR products**	**Primer (genotyping)**	**Sequence**
Cx3cr1^{CreERT2}<br>Mutant = ~300 bp<br>Heterozygote = ~300 bp and 695 bp<br>Wild type = 695 bp	common forward	AAGACTCACGTGGACCTGCT
	mutant reverse	CGGTTATTCAACTTGCACCA
	wild type reverse	AGGATGTTGACTTCCGAGTTG
CD47-KO<br>Mutant = 199 bp<br>Heterozygote = 199 bp and 300 bp<br>Wild type = 300 bp	common forward	GAAGTGGAAGTTGAACAAATCG
	mutant reverse	TGGGCTCTATGGCTTCTGAG
	wild type reverse	CACCTTACAGCACTCCCACA
APP/PS1<br>Mutant = 142 bp<br>Heterozygote = 142 bp and 265 bp<br>Wild type = 265 bp	wild type forward	GTGTGATCCATTCCATCAGC
	mutant forward	ATGGTAGAGTAAGCGAGAACACG
	common reverse	GGATCTCTGAGGGGTCCAGT
SIRPα fl/fl<br>Mutant = 406 bp<br>Heterozygote = 340 bp and 406 bp<br>Wild type = 340 bp	SIRPα fl 5' forward	TTTGACAGTGCAGCAACACCAGG
	SIRPα fl 5' reverse	ATCCTTGCCACCCAAGCACAGTGG
SIRPα-cKO after tamoxifen induction<br>Mutant = 343 bp<br>Heterozygote = 343 bp<br>Wild type, no products (14633 bp)	forward	TTTGACAGTGCAGCAACACCAGG
	reverse	ATGGCTGTGCTTCCACT CTCACTT
**Gene**	**Primer (quantification)**	
18s RNA	forward	ATCCATTGGAGGGCAAGTCT
	reverse	CCGCGGTCCTATTCCATTAT
SIRPβ1	forward	CCCGTTCACAGGAGAACATT
	reverse	CCGGAGACCATAGGTGAAGA
