# Supplementary material for: Loss of microglial SIRPα promotes synaptic pruning in preclinical models of neurodegeneration
Source: Nat Commun. 2021 Apr 1;12:2030. doi: 10.1038/s41467-021-22301-1 (PMC8016980; doi:10.1038/s41467-021-22301-1)
Supplement: Supplementary file 4 — Reporting Summary [file 41467_2021_22301_MOESM4_ESM.pdf]

nature research

Corresponding author(s): Liang Li, Ke Zen

Last updated by author(s): Mar 1, 2021

# Reporting Summary

Nature Research wishes to improve the reproducibility of the work that we publish. This form provides structure for consistency and transparency in reporting. For further information on Nature Research policies, seeAuthors & Referees and theEditorial Policy Checklist .

## Statistics

For all statistical analyses, confirm that the following items are present in the figure legend, table legend, main text, or Methods section.

| n/a | Confirmed | |
|---|---|---|
| ☐ | ☒ | The exact sample size (*n*) for each experimental group/condition, given as a discrete number and unit of measurement |
| ☐ | ☒ | A statement on whether measurements were taken from distinct samples or whether the same sample was measured repeatedly |
| ☐ | ☒ | The statistical test(s) used AND whether they are one- or two-sided<br>*Only common tests should be described solely by name; describe more complex techniques in the Methods section.* |
| ☒ | ☐ | A description of all covariates tested |
| ☐ | ☒ | A description of any assumptions or corrections, such as tests of normality and adjustment for multiple comparisons |
| ☐ | ☒ | A full description of the statistical parameters including central tendency (e.g. means) or other basic estimates (e.g. regression coefficient) AND variation (e.g. standard deviation) or associated estimates of uncertainty (e.g. confidence intervals) |
| ☐ | ☒ | For null hypothesis testing, the test statistic (e.g. *F*, *t*, *r*) with confidence intervals, effect sizes, degrees of freedom and *P* value noted<br>*Give P values as exact values whenever suitable.* |
| ☒ | ☐ | For Bayesian analysis, information on the choice of priors and Markov chain Monte Carlo settings |
| ☒ | ☐ | For hierarchical and complex designs, identification of the appropriate level for tests and full reporting of outcomes |
| ☒ | ☐ | Estimates of effect sizes (e.g. Cohen's *d*, Pearson's *r*), indicating how they were calculated |

*Our web collection on statistics for biologists contains articles on many of the points above.*

## Software and code

Policy information about availability of computer code

| | |
|---|---|
| Data collection | All fluorescence images were captured on a Leica TCS SP8 MP confocal microscope (Leica Microsystems) with the LAS X 3.3.0 software, a confocal microscope (Zeiss LSM880) with ZEN 3.1 blue edition software, or an Olympus microscope BX51 (Olympus) with Cell sens dimension 1.12 software(Olympus). Flow cytometry was carried out on a Gallios flow Cytometer (Beckman Coulter) with Kaluza 1.0 software (Beckman Coulter) . Synaptosome data were collected by NanoSight NS300(Malvern Instruments) through NanoSight NTA 3.2 software(Malvern Instruments). Behavioral data were collected by DigBehv-MM tracker system (MobileDatum Co. Ltd, Shanghai, China). mEPSCs were recorded through Clampex 10.7(Axon Instruments). |
| Data analysis | For quantification of western blots and the immunofluorescence signals, we used ImageJ-Fiji 2.0.0 software(NIH, Bethesda, MD). Flow cytometry was analysed by Kaluza Analysis 1.5a software(Beckman Coulter). 3D volume surface renderings of each z-stack was created using Imaris 7.4.2 software (Bitplane).mEPSC data were analysed by pClamp10.0 (Axon Instruments). For statistical analyses, we used GraphPad PRISM 8 software and GPower 3.1 software(Heinrich-Heine-Universität Düsseldorf). |

For manuscripts utilizing custom algorithms or software that are central to the research but not yet described in published literature, software must be made available to editors/reviewers. We strongly encourage code deposition in a community repository (e.g. GitHub). See the Nature Research guidelines for submitting code & software for further information.

## Data

Policy information about availability of data

All manuscripts must include a data availability statement. This statement should provide the following information, where applicable:

- Accession codes, unique identifiers, or web links for publicly available datasets
- A list of figures that have associated raw data
- A description of any restrictions on data availability

All relevant data generated for this study are included in the article/Supplementary Material/Source Data File. Other data/materials (including SIRPα fl/fl mice) that support the findings of this study are readily available from the corresponding author upon reasonable request. (Applicants should sign the material transfer agreement and take charge of all the cost regarding to mouse transfer).

# Field-specific reporting

Please select the one below that is the best fit for your research. If you are not sure, read the appropriate sections before making your selection.

- [x] Life sciences
- [ ] Behavioural & social sciences
- [ ] Ecological, evolutionary & environmental sciences

For a reference copy of the document with all sections, see nature.com/documents/nr-reporting-summary-flat.pdf

# Life sciences study design

All studies must disclose on these points even when the disclosure is negative.

| | |
|---|---|
| Sample size | Using G-power software 3.1, sample size is estimated by the significance level (0.05), effect size (base on the data in our previous study) and a given power (usually 0.8). We also conducted power analysis after the experiment and found enough power value (>0.8) in each statistical analysis. |
| Data exclusions | No data were excluded from the analyses. |
| Replication | The experimental findings were reproduced in multiple independent experiments. The number of independent experiments and biological replicates in each data panel is indicated in the figure legends. |
| Randomization | Mice/cells were assigned randomly into experimental groups and processed in an arbitrary order. |
| Blinding | All the enrolled mice or subsequent samples were labelled only with mouse ID numbers and did not indicate genotype or type of treatment. Genotype or treatment type were decoded after the data acquisition and quantification analysis were complete. |

# Reporting for specific materials, systems and methods

We require information from authors about some types of materials, experimental systems and methods used in many studies. Here, indicate whether each material, system or method listed is relevant to your study. If you are not sure if a list item applies to your research, read the appropriate section before selecting a response.

## Materials & experimental systems

| n/a | Involved in the study | |
|---|---|---|
| | x | Antibodies |
| x | | Eukaryotic cell lines |
| x | | Palaeontology |
| | x | Animals and other organisms |
| x | | Human research participants |
| x | | Clinical data |

## Methods

| n/a | Involved in the study | |
|---|---|---|
| x | | ChIP-seq |
| | x | Flow cytometry |
| x | | MRI-based neuroimaging |

## Antibodies

Antibodies used

anti-beta-Amyloid (clone 6E10), 1:500, BioLegend Cat# 803001, RRID:AB_2564653
CD16/CD32 antibody(clone KT1632) 1:200 Thermo Fisher Scientific Cat#MA5-18012,RRID:AB_2539396
CD47 antibody (western blot) 1:1000 R and D Systems Cat# AF1866, RRID:AB_2074942
CD47 antibody(immunofluorescence) 1:200 BD Pharmingen Cat#555297, RRID:AB_395713
GAPDH antibody (Clone 2d4a7), 1:2000, Santa Cruz Biotechnology Cat# sc-59541, RRID:AB_783594
GFAP antibody 1:1000 Abcam Cat# ab4674, RRID:AB_304558
Homer antibody, 1:200 Synaptic Systems Cat#160-003; RRID:AB _887730
Iba-1 antibody, 1:500, Wako Cat# 019-19741 RRID:AB_839504

MAP2 antibody, 1:1000 Abcam Cat# ab5392, RRID:AB_2138153
NeuN antibody [EPR12763] 1:500 Abcam Cat# ab177487,RRID:AB_2532109
PSD95 (D27E11) antibody (rabbit origin), 1:500 Cell Signaling Technology Cat#3450, RRID:AB_2292883
PSD95 antibody[6G6-1C9] (mouse origin) 1:100-1:200 Abcam Cat#ab2723,RRID:AB_303248
SIRPα antibody (western blot), 1:5000, Abcam Cat# ab8120, RRID:AB_882558
SIRPα (clone OX-41) antibody(immunofluorescence) 1:500 Millipore Cat# MAB1407P;
SIRPB1/CD172b antibody 1:2000 LifeSpan Biosciences, Cat# LS-C679465
SynapsinI antibody, 1:1000 Millipore, Cat#AB1543
Synaptophysin antibody(SVP-38), 1:2000 Sigma-Aldrich Cat# S5768, RRID:AB_477523
Vglut1 antibody, 1:1000 Millipore Cat#AB5905; RRID:AB_2301751
Vglut2 antibody, 1:1000 Millipore Cat#AB2251, RRID:AB_2665454

CD11b Antibody (M1/70)- APC, 1:200 eBioscience Cat# 17-0112-82, RRID:AB_469343
CD45 Antibody (30-F11)-PE, 1:200 eBioscience Cat# 12-0451-82, RRID:AB_465668
CD47 Antibody-FITC, 1:100 BD Pharmingen Cat# 555298, RRID:AB_395714
CD172a (SIRPα) Antibody-FITC 1:200 BioLegend Cat#144006, RRID: AB_11204425
CX3CR1 antibody- Alexa Fluor 647 1:100 BioLegend Cat# 149004, RRID:AB_2564273

Donkey anti-rabbit IgG-HRP 1:1000-1:5000 antibody Santa Cruz Biotechnology Cat# sc-2077, RRID:AB_631745
Donkey anti-mouse IgG-HRP antibody 1:2000-1:5000 Santa Cruz Biotechnology Cat# sc-2314, RRID:AB_641170
Donkey anti-goat IgG-HRP antibody 1:1000-1:2000 Santa Cruz Biotechnology Cat# sc-2020, RRID:AB_631728

Goat anti-Rabbit IgG (H+L) Secondary Antibody, Alexa Fluor 488 Thermo Fisher Scientific Cat# A-11034, RRID:AB_2576217
Goat Anti-Rabbit IgG (H+L) Secondary Antibody, Alexa Fluor 594 Thermo Fisher Scientific Cat# A-11012, RRID:AB_141359
Goat anti-Rabbit IgG (H+L) Secondary Antibody, Alexa Fluor 633 Thermo Fisher Scientific Cat# A-21071, RRID:AB_2535732
Goat anti-Mouse IgG (H+L) Secondary Antibody, Alexa Fluor 488 Thermo Fisher Scientific Cat# A-11001, RRID:AB_2534069
Goat anti-Mouse IgG (H+L) Secondary Antibody, Alexa Fluor 594 Thermo Fisher Scientific Cat# A-11005, RRID:AB_141372
Goat anti-Chicken IgY (H+L) Secondary Antibody, Alexa Fluor 488 Thermo Fisher Scientific Cat# A-11039, RRID:AB_2534096
Goat anti-Chicken IgY (H+L) Secondary Antibody, Alexa Fluor 594 Thermo Fisher Scientific Cat# A-11042, RRID:AB_2534099
Donkey Anti-Rat IgG (H+L) Secondary Antibody, Alexa Fluor 594 Thermo Fisher Scientific Cat# A-21209, RRID:AB_2535795
Donkey anti-Rat IgG (H+L) Secondary Antibody, Alexa Fluor 488 Thermo Fisher Scientific Cat# A-21208, RRID:AB_2535794
Goat Anti-Guinea Pig IgG (H+L) Secondary Antibody, Alexa Fluor 488 Thermo Fisher Scientific Cat# A-11073, RRID:AB_142018
CD11b (Microglia) MicroBeads, human and mouse Miltenyi Biotec Cat# 130-093-634
Human beta-Amyloid(1-42) ELISA Kit Wako, High-Sensitive, Wako Cat#296-64401

Validation

All antibodies have been tested for reactivity against the appropriate species on the specification sheets on the providers' websites or in published articles.
According to the manufacturer's website, anti-beta-Amyloid (clone 6E10) antibody (BioLegend, 803001) is suitable for western blot, ELISA, immunohistochemistry and immunocytochemistry, and reacts with human. This antibody was previously validated in Yan Zheng et al., 2012, Plos one.
According to the manufacturer's website, CD16/32(clone KT1632) antibody (Thermo Fisher Scientific, MA5-18012) is suitable for flow cytometry, and reacts with mouse. Flow cytometric analysis of peritoneal macrophages staining by CD32/CD16 antibody is provided on the website.
According to the manufacturer's website, CD47 antibody (R and D Systems, AF1866) is suitable for western blot, and reacts with mouse and rat. Western blot examples of mouse CD47 is provided on the website.
According to the manufacturer's website, CD47 antibody (BD PharMingen, 555297) is suitable for immunohistochemistry, fluorescence microscopy and western blot, reacts with mouse. This antibody was previously validated in Lehrman et al., 2018, Neuron.
According to the manufacturer's website, GAPDH antibody (Santa Cruz Biotechnology, sc-59541) is suitable for western blot and immunoprecipitation, reacts with mouse and human. Western blot examples of GAPDH is provided on the website.
According to the manufacturer's website, GFAP antibody (Abcam, ab4674) is suitable for immunohistochemistry, immunocytochemistry and western blot, reacts with mouse and rat, immunohistochemistry example of mouse brain slice is provided on the website.
According to the manufacturer's website, Homer antibody (Synaptic Systems, 160-003) is suitable for western blot, immunoprecipitation, immunocytochemistry, immunohistochemistry and ELISA, and reacts with human, rat and mouse. This antibody was previously validated in Lehrman et al., 2018, Neuron.
According to the manufacturer's website, Iba-1 antibody is suitable for immunohistochemistry, and reacts with mouse. This antibody was previously validated in Lehrman et al., 2018, Neuron.
According to the manufacturer's website, MAP2 antibody (Abcam, ab5392) is suitable for immunocytochemistry and western blotting, reacts with mouse and rat. Immunocytochemistry example of mouse neuron is provided on the website.
According to the manufacturer's website, NeuN antibody (Abcam, ab177487) is suitable for immunohistochemistry in mouse and human. Immunohistochemistry example of mouse brain slice is provided on the website.
According to the manufacturer's website, anti-PSD95 antibody (Abcam, ab2723) is suitable for immunohistochemistry and western blot, reacts with mouse and rat. This antibody was previously validated in Qiong Wu et al., 2020, Front Aging Neurosci.
According to the manufacturer's website, PSD95 antibody (Cell signaling technology, 3450) is suitable for western blot and immunofluorescence, and reacts with human, mouse and rat. This antibody was previously validated in Illendula et al., 2020, Front Aging Neurosci.
According to the manufacturer's website, SIRPα antibody (Abcam, ab8120) is suitable for western blot and

immunohistochemistry, reacts with human, and this antibody has been reported mouse reactivity in Jin Wang et al., 2019, Glia.
According to the manufacturer's website, SIRPα antibody (Millipore, MAB1407P) is suitable for immunohistochemistry, immunocytochemistry, flow Cytometry and western blot. It reacts with human, mouse and rat. It was previously validated in Jin Wang et al., 2019, GLIA.
According to the manufacturer's website, SIRPβ1 antibody (LifeSpan Biosciences, C679465) is suitable for ELISA, Western blotting, immunohistochemistry and immunofluorescence, reacts with mouse and human. Western blot examples of SIRPβ are provided on the website.
According to the manufacturer's website, SynapsinI (Millipore, AB1543) antibody is suitable for immunocytochemistry, immunohistochemistry, immunoprecipitation and western blot, and reacts with bovine, human, mouse, rat. This antibody was previously validated in Yuanchao Xue et al., 2013, Cell.
According to the manufacturer's website, Synaptophysin antibody (Sigma-Aldrich, S5768) is suitable for immunohistochemistry, indirect ELISA and western blotting. It reacts with human, guinea pig, rat and pig. In Lui et al., 2016, Cell., Synaptophysin antibody was reported mouse reactivity for primary cell staining.
According to the manufacturer's website, Vglut1 antibody (Millipore, AB5905) is suitable for immunohistochemistry, and reacts with rat. Vglut1 antibody has been reported mouse reactivity in Lehrman et al., 2018, Neuron.
According to the manufacturer's website, Vglut2 antibody (Millipore, AB2251) is suitable for immunohistochemistry and western blot, and reacts with rat and mouse. This antibody was previously validated in Lehrman et al., 2018, Neuron.
According to the manufacturer's website, CD11b-APC antibody (eBioscience, 17-0112-82) is suitable for flow cytometry, reacts with mouse. The website provided a flow cytometric figure in which mouse bone marrow cells were staining with isotype control or CD11b APC to show the validation.
According to the manufacturer's website, CD45-PE antibody (eBioscience,12-0451-82) is suitable for flow cytometry, and reacts with mouse. The website provided flow cytometric figures of mouse cells to verify the validation.

According to the manufacturer's website, CD47-FITC (Clone miap301) antibody (BD Pharmingen, 555298) is suitable for flow cytometry, and reacts with mouse. This antibody was previously validated in C. Treese et al., 2008, Cytometry A.
According to the manufacturer's website, SIRPα-FITC antibody (BioLegend, 144006) is suitable for flow cytometry, and reacts with mouse. The website verified the validation by providing a flow cytometric figure in which mouse bone marrow cells were stained with CD11b APC and CD172a.
According to the manufacturer's website, CX3CR1-647 antibody (BioLegend, 149004) is suitable for flow cytometry and reacts with mouse. CX3CR1-647 antibody has been reported immunofluorescence application in Lehrman et al., 2018, Neuron.

## Animals and other organisms

Policy information about studies involving animals; ARRIVE guidelines recommended for reporting animal research

Laboratory animals

Cx3cr1CreERT2 mice, CD47 KO mice and AD (APPswe, PSEN1dE9) mice were purchased from the Model Animal Research Center of Nanjing University. SIRPα fl/fl mice were generated by flanking sirpα exon 2-6 with loxP sites. All animals are maintained on a C57BL/6 background. Microglial SIRPα-cKO mice were generated after we treated Cx3cr1CreERT2: SIRPα fl/fl mice with tamoxifen (TAM) at different stages. P5,P10, P15,P28,P30, P60, P90, 5-months old,6-months old, 8-months old,12-months old mice were used as described in the manuscript. SIRPα-cKO mice were treated by TAM at P1,P2,P3, then sacrificed for further analyses at P5,P10, P15, P28, P30. SIRPα-cKO/AD mice were treated by TAM at 2-months old, and were tested at 5/8 months old. SIRPα-cKO mice were treated by TAM at 2-months old, and subjected to Abeta at P90, 3-10 days later mice were sacrificed for analyses. All animals used in this study are male.

Wild animals

none

Field-collected samples

none

Ethics oversight

All experimental procedures were approved by Model Animal Research Center of Nanjing University and according with Laboratory Animal Care Guidelines.

Note that full information on the approval of the study protocol must also be provided in the manuscript.

## Flow Cytometry

### Plots

Confirm that:

- [x] The axis labels state the marker and fluorochrome used (e.g. CD4-FITC).
- [x] The axis scales are clearly visible. Include numbers along axes only for bottom left plot of group (a 'group' is an analysis of identical markers).
- [x] All plots are contour plots with outliers or pseudocolor plots.
- [x] A numerical value for number of cells or percentage (with statistics) is provided.

### Methodology

Sample preparation

Mice were anesthetic and executed to harvest cerebrums. Cerebrums were dissected into small pieces and digested in papain for 30 min. After digestion, cell suspensions were filtered through 70 μm cell strainer to remove clumps, and cell pellets were

| | |
|---|---|
| | resuspended in 30% Percoll solution following centrifugation. The upper myelin layer was discarded, and cell pellets were resuspended in medium (HBSS containing 2% FBS and 1 mM EDTA) to obtain single-cell suspension. |
| Instrument | Beckman Coulter Gallios flow cytometry. |
| Software | Kaluza for Gallios acquisition software(Beckman). |
| Cell population abundance | No cells were sorted from cell population and no post-sort analysis was done. Cell clumps and debris were excluded. |
| Gating strategy | CD45 int CD11b high cells (population in pentagon in S figure1f) were considered as microglia. |

☒ Tick this box to confirm that a figure exemplifying the gating strategy is provided in the Supplementary Information.
